# Synergistic effect of bioactive lipid and condition medium on cardiac differentiation of human mesenchymal stem cells from different tissues

**DOI:** 10.1002/cbf.3175

**Published:** 2016-03-16

**Authors:** Lili Jiang, Yanwen Wang, Fang Pan, Xiubo Zhao, Henggui Zhang, Ming Lei, Tianqing Liu, Jian R. Lu

**Affiliations:** ^1^ Dalian R&D Center for Stem Cell and Tissue Engineering, Faculty of Chemical Environmental and Biological Science and TechnologyDalian University of TechnologyDalianChina; ^2^ Biological Physics Group, School of Physics and AstronomyUniversity of ManchesterManchesterUK; ^3^ Cardiovascular and Genetic Medicine Research Groups, School of BiomedicineUniversity of ManchesterManchesterUK; ^4^ Department of Chemical & Biological EngineeringUniversity of SheffieldMappin Street, Sheffield, S1 3JDUK

**Keywords:** cardiac differentiation, synergistic effect, bioactive lipid, calcium transients, 5‐azacytidine

## Abstract

Human umbilical cord mesenchymal stem cells (hUCMSCs) and human adipose tissue mesenchymal stem cells (hATMSCs) have the potential to differentiate into cardiomyocytes, making them promising therapeutic candidates for treating damaged cardiac tissues. Currently, however, the differentiated cells induced from hUCMSCs or hATMSCs can hardly display functional characteristics similar to cardiomyocytes. In this study, we have investigated the effects of bioactive lipid sphingosine‐1‐phosphate (S1P) on cardiac differentiations of hUCMSCs and hATMSCs in condition medium composed of cardiac myocytes culture medium or 5‐azacytidine. Cardiac differentiations were identified through immunofluorescence staining, and the results were observed with fluorescence microscopy and confocal microscopy. Synergistic effects of S1P and condition medium on cell viability were evaluated by MTT assays. Functional characteristics similar to cardiomyocytes were evaluated through detecting calcium transient. The differentiated hUCMSCs or hATMSCs in each group into cardiomyocytes showed positive expressions of cardiac specific proteins, including α‐actin, connexin‐43 and myosin heavy chain‐6 (MYH‐6). MTT assays showed that suitable differentiation time was 14 days and that the optimal concentration of S1P was 0.5 μM. Moreover, incorporation of S1P and cardiac myocytes culture medium gave rise to calcium transients, an important marker for displaying *in vivo* electrophysiological properties. This feature was not observed in the S1P‐5‐azacytidine group, indicating the possible lack of cellular stimuli such as transforming growth factor‐beta, TGF‐β. © 2016 The Authors. Cell Biochemistry and Function published by John Wiley & Sons, Ltd.

## Introduction

Mesenchymal stem cells (MSCs) have the ability to differentiate into various types of tissue cells. This property is of enormous potential to clinical applications in tissue regeneration covering bone, cartilage, muscle and adipose.^1^, ^2^, ^3^ A number of recent reports have shown that MSCs could also differentiate into cardiomyocytes *in vivo* and *in vitro*.^4^, ^5^

Two types of MSCs, human umbilical cord mesenchymal stem cells (hUCMSCs) and human adipose tissue mesenchymal stem cells (hATMSCs), have been confirmed to possess the characteristics similar to MSCs from other sources. Therefore, hUCMSCs or hATMSCs have the potential to differentiate into cardiomyocytes and express cardiac specific proteins, such as sarcomeric α‐actinin, cardiac troponin I, β‐myosin heavy chain and connexin‐43.^6^, ^7^, ^8^, ^9^, ^10^ On the basis of these findings, hUCMSCs or hATMSCs have become promising therapeutic candidates for treating damaged cardiac tissues.^8^, ^11^, ^12^ However, the present differentiated cells from hUCMSCs or hATMSCs that are induced simply from cardiac myocytes culture medium (CMCM) or 5‐azacytidine hardly display functional characteristics similar to cardiomyocytes, such as contractile activity or calcium transient. On the other hand, microenvironmental factors such as mechanical cues, soluble bioactive factors and electricity stimulation cannot only regulate MSC differentiations but also modulate MSC signalling to the surrounding environment.^13^ Therefore, it is important to find a bioactive factor combining with cardiac differentiation medium for hUCMSCs or hATMSCs to improve their functional cardiomyogenic differentiation.

Sphingosine‐1‐phosphate (S1P) is a pleiotropic bioactive lipid capable of regulating diverse biological processes, including cell growth, cell survival, cell migration and cell differentiation.^14^, ^15^, ^16^ A recent study has reported that S1P dose‐dependently stimulated the differentiation of hATMSCs towards smooth muscle cells.^13^ A previous work has also shown that S1P can promote the differentiation of hUCMSCs towards functional cardiomyocytes under CMCM.^17^ These studies have provided us clues of possible induction of hUCMSCs under S1P into cardiomyocytes. Furthermore, it is useful to find out whether S1P can promote MSCs derived from other tissues such as hATMSCs to differentiate towards functional cardiomyocytes under CMCM and 5‐azacytidine medium, also a popular induction medium.

Therefore, in this study we have examined the induction of both hUCMSCs and hATMSCs with S1P and 5‐azacytidine to investigate their effects on the cardiomyogenic differentiation of the two MSCs *in vitro*. In addition, we explored the induction time and the optimal concentration of S1P by evaluating cell viability during the induction processes.

## Materials and Methods

### Cell culture and harvest

Human cardiac myocytes (HCM, Cat. No. 6200) and hUCMSCs (Cat. No. 6252) were purchased from ScienCell Research Laboratories (San Diego, CA, USA). Human adipose tissue was obtained from five healthy female donor patients aged 18 to 45 undergoing elective liposuction with informed patient consents. The hATMSCs were isolated as reported previously.^18^ Briefly, lipoaspirates were digested using digestion buffer which was comprised of 0.1% collagenase type I and 0.25% trypsin (Sigma, v/v 1:1) and incubated at 37 °C with constant agitation for 20 min. Following digestion, Dulbecco's Modified Eagle Medium (DMEM, Sigma) containing 10% fetal bovine serum (FBS, Sigma) was added to inactivate the digestion buffer and the mixture was centrifuged at 1500 rpm for 10 min. The cellular pellet was resuspended in culture medium (DMEM + 10% FBS) and plated in T flasks.

For cultivation, the cells were kept in DMEM containing 10% FBS, 2 mM glutamine (Sigma, USA), 100 units/ml penicillin (Sigma, USA) and 100 µg ml^−1^ streptomycin (Sigma, USA) and were incubated at 37 °C under a humidified atmosphere containing 5% CO_2_. Once the culture reached over 90% confluence, the cells were detached with trypsin/EDTA (Sigma, USA) and collected by centrifuging at 1000 rpm for 5 min. The cells were plated into flasks or dishes at 5000 cells cm^−2^ for subculturing. Cultures after passage 4 (P4) were used in this study.

### Cardiac myocyte culture medium collection

To collect the CMCM, HCM cells were seeded into the flasks at 5000 cells cm^−2^. After 24 h of seeding, the culture medium was collected and new culture medium was replaced every day.

### Cardiomyogenic

hUCMSC or hATMSC differentiations into cardiomyocytes were induced using a combination of chemicals (5‐azacytidine and S1P, both from Sigma) and cardiac microenvironment (CMCM). The induction experiments were divided into six groups: MSCs cultured in CMCM (group A), in CMCM + 0.1 μM S1P (group B), in CMCM + 0.5 μM S1P (group C), in CMCM + 1.0 μM S1P (group D), in DMEM + 5‐azacytidine (group E), in DMEM + 5‐azacytidine + 0.5 μM S1P (group F) and two control groups (hUCMSCs or hATMSCs cultured in DMEM as negative control, HCM cultured in DMEM as positive control). hATMSCs and hUCMSCs after P4 were seeded into 24‐well plates at 5000 cells cm^−2^ and induced to differentiate into cardiomyocytes after 3 days of plating with the above media.

In 5‐azacytidine groups (group E and group F), 10 μM of 5‐azacytidine was used. After incubating for 24 h in DMEM, the cells were washed with phosphate buffer solution (PBS, Sigma), and the media were changed to differentiation media. Then the media was changed every 3 days. Cell differentiation was evaluated at the 7th day, the 14th day and the 28th day after the drug treatment by immunofluorescence staining of cardiac specific proteins.

In CMCM groups (groups A–D), the induction media (CMCM with different concentrations of S1P at 0, 0.1, 0.5 and 1 μM) were used. Each medium was changed every 3 days. Cell differentiations were evaluated at the 7th, 14th and 28th day after the drug treatment by immunofluorescence staining of cardiac specific proteins.

### Immunofluorescence staining of specific proteins

To identify whether hATMSCs and hUCMSCs induced in 5‐azacytidine groups and CMCM groups became differentiated into cardiomyocytes, immunofluorescence staining of three cardiomyocyte proteins was performed: α‐actin, connexin‐43 and MYH‐6 (myosin heavy chain‐6). Firstly, the cells were cultured and induced on glass cover slips. After induction, the cells were washed three times with PBS and fixed with 4% paraformaldehyde at the room temperature for 15 min. After being washed three times again with PBS, the fixed cells were permeabilized for 30 min in PBS containing 0.1% Triton X‐100, and then blocked with 10% normal goat serum in PBS for an hour at 37 °C. Then the cells were incubated in rabbit polyclonal primary antibodies against α‐actin, connexin‐43 and MYH‐6, respectively, at 4 °C overnight. The cells were subsequently incubated in FITC‐conjugated secondary antibodies at the room temperature for 1 h. Negative controls and positive controls were also performed to offset the disturbance of the primary or secondary antibodies. The cells on the glass cover slips were counterstained with DAPI (Sigma) to visualize their nuclei. The results were observed with fluorescence microscopy and confocal microscopy, and the images were analysed using software of Image J.

### Cell activity assay

To compare the cell activities under different induction conditions, hATMSCs and hUCMSCs were seeded in 6‐well plates in growth medium (DMEM + 10% FBS) first. After 3 days of culture, the growth medium was changed into induction medium containing different concentration of S1P. The cell activities in different induction groups were measured using MTT assays at 7th day and 14th day, respectively. Three hundred microlitres of MTT solution (5 mg ml^−1^ in PBS) was added into each well of 6‐well plate and incubated at 37 °C for 4 h. Then, the MTT solution was aspirated from the wells, and 3 ml of dimethyl sulfoxide (DMSO) was added to solubilize the formazan crystals. After incubation of 10 min, 200 μL of DMSO solution was then transferred to new wells of 96‐well plate to facilitate the absorbance measurement at 570 nm. All measurements were performed in triplicate.

### Electrophysiological measurement

To compare the electrophysiological features of the cells under different induction conditions, hATMSCs and hUCMSCs were seeded in 6‐well plates in growth medium (DMEM + 10% FBS) first. After 3 days of culture, the growth medium was changed into induction medium including group A (CMCM), group C (CMCM + 0.5 μM S1P), group E (5‐azacytidine) and group F (5‐azacytidine + 0.5 μM S1P). The electrophysiological features of the cells in different induction groups were measured using the patch clamp technique at the 14th day.

For electrophysiological recordings, the cells were plated on the 9 mm diameter round cover slips in dishes containing DMEM and left for one day before the experiments. For electrical field stimulation, the cover slips were placed in the middle of a chamber under microscope and perfused with 37 °C pre‐warmed DMEM for 5 min. Then 5 μL of the Fluo‐3 calcium indicator (Invitrogen) was added into the chamber with both in‐flow and out‐flow being shut. After incubation of 10 min, the cells were continuously perfused with 37 °C pre‐warmed DMEM for at least 40 min to allow the de‐esterification. The cells were then exposed under fluorescence at 488 nm, the excitation wavelength of Fluo‐3. Any calcium transient induced by the stimulation would be observed by 40× oil immersion objective and recorded by pCLAMP programme 10.0 (Axon Instrument Inc, 1101 Chess Drive, Foster City, CA, USA). The stimulation was performed by applying rectangular pulses of alternate polarity with 2 ms duration and 200 mA amplitude. For electrophysiological recordings, the cells were stimulated with constant pulses at 1 Hz frequency by Spike 2 programme. Electrical signals were digitized at 5 kHz by a DigiData 1322A A/D converter (Axon Instruments Inc) and stored on a computer for later analysis.

### Statistical analysis

All the experiments were repeated three times, and the data were averaged from three separate experiments. The data were analysed by OriginPro8.0 software (OriginLab Corporation, USA) and expressed as mean ± standard deviation (SD, *n* = 3). The Student's *t*‐tests were used to compare differences between the two groups in differentiations of hATMSCs and hUCMSCs into cardiomyocytes and cell viability, with *p* < 0.05 being considered significant.

## Results

### Influence of induction time on differentiation

To offset various disturbances including influences from the primary or secondary antibodies, negative and positive controls were performed. hUCMSCs or hATMSCs were cultured in normal medium (DMEM) as negative controls, while HCM cells were cultured in cardiomyogenic medium (DMEM) as positive control. The results of the two negative controls showed that hUCMSCs (Figure S1B) and hATMSCs (Figure S1C) had no expression of the three cardiac specific proteins (α‐actin, connexin‐43 and MYH‐6), while HCM cells expressed all of them (Figure S1A).

**Figure 1:**
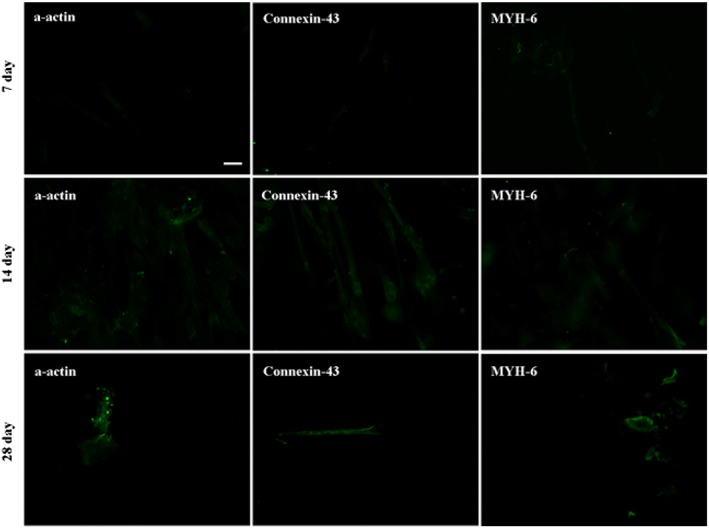
Influence of induction time of S1P on the cardiogenic differentiation of hUCMSCs. The expressions of the three cardiac specific proteins were used to evaluate the cardiomyogenic differentiations of hUCMSCs. The results of group C (CMCM + 0.5 μM S1P) were presented. On the 7th day, the cells began to express α‐actin, connexin‐43 and MYH‐6, but the fluorescence intensities were weak. At the 14th day, most of the cells expressed three specific proteins and the fluorescence intensities were stronger than those on the 7th day. The expressions of the cardiac specific proteins on the 28th day were similar to those on the 14th day, but then most of the cells were dead and only a small number of them remained alive. The bars are 100 µm

**Figure 2:**
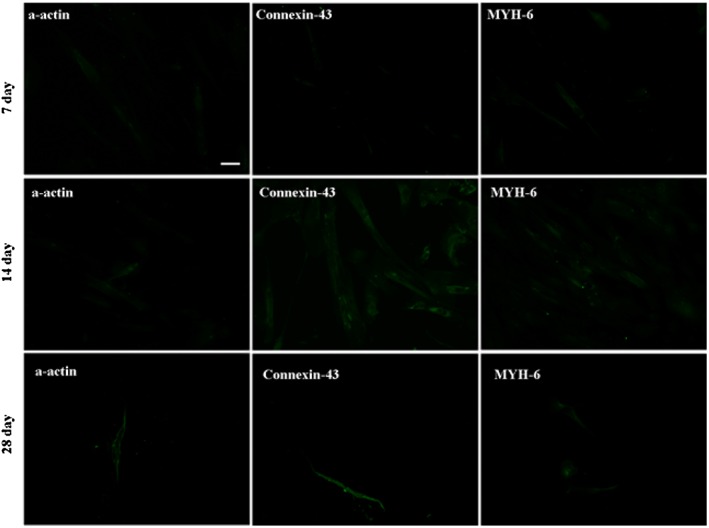
Influence of induction time of S1P on the cardiogenic differentiation of hATMSCs. The expressions of the three cardiac specific proteins were used to evaluate the cardiomyogenic differentiations of hATMSCs. The results of group D (CMCM + 1.0 μM S1P) were presented. On the 7th day, the cells began to express α‐actin, connexin‐43 and MYH‐6, but the fluorescence intensities were weak. At the 14th day, most of the cells expressed three specific proteins and the fluorescence intensities were stronger than those on the 7th day. The expressions of cardiac specific proteins on the 28th day were similar to those on the 14th day, but most of the cells were dead and only a small number of them remained alive. The bars are 100 µm

Differentiations of hATMSCs and hUCMSCs in each group into cardiomyocytes were then undertaken and evaluated by immunofluorescence staining of cardiac specific proteins (α‐actin, connexin‐43 and MYH‐6) on the 7th, 14th and 28th day, respectively. As a representative example, the results of groups C and D are shown in Figures 1 and 2, with those of the control groups being presented in Figure S1. It can be seen that both hUCMSCs (Figure 1) and hATMSCs (Figure 2) expressed the three cardiac specific proteins after 7 days of induction under the influence of CMCM and S1P (0.5 and 1 μM) , but the fluorescence intensities were weak at this time. After 14 days of incubation, most of the cells continued to express the three cardiac specific proteins and the fluorescence intensities became stronger than those at the 7th day. On the 28th day, the expressions of the three cardiac specific proteins were similar to those obtained on the 14th day in intensity, but most of the cells became dead and only a small number of them were left, indicating stronger expressions of the three proteins from the surviving cells. These results indicated that the suitable differentiation time was likely to be around day 14 taking into account the balance between protein expression and cell survival rate. Thus, the optimal induction time was set at day 14 in the subsequent experiments.

### Influence of S1P concentration on differentiation

**Figure 3:**
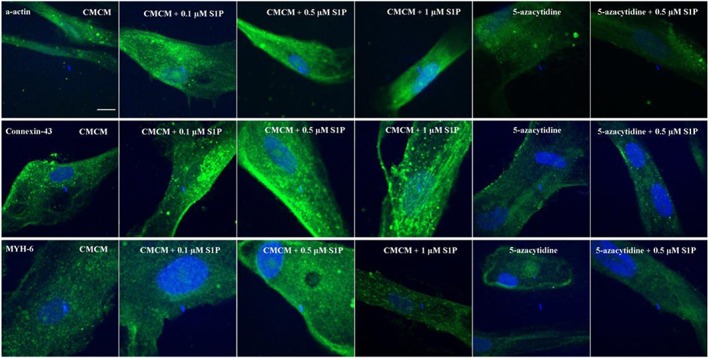
Influence of S1P concentration on the differentiation of hUCMSCs toward cardiomyocytes in induction media. This experiment was divided into six groups, including CMCM (group A), CMCM + 0.1 μM S1P (group B), CMCM + 0.5 μM S1P (group C), CMCM + 1.0 μM S1P (gorup D), DMEM + 5‐azacytidine (group E) and DMEM + 5‐azacytidine + 0.5 μM S1P (group F). The differentiations were examined by immunofluorescence staining of cardiac specific proteins, and the results were evaluated by confocal microscopy. All the differentiated cells in the six groups expressed three cardiac specific proteins (α‐actin, connexin‐43 and MYH‐6), but the fluorescence intensities from these expressions were different. The bars are 20 µm

**Figure 4:**
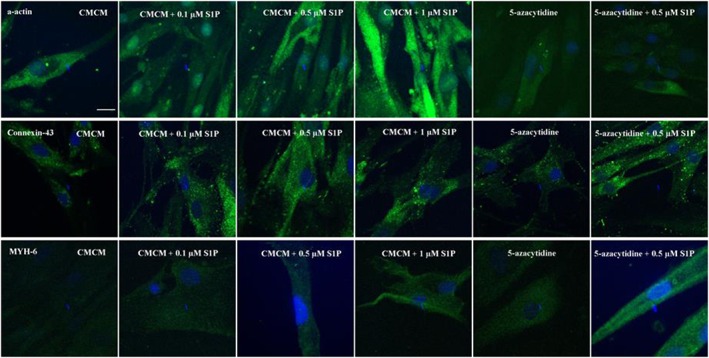
Influence of S1P concentration on the differentiation of hATMSCs toward cardiomyocytes in induction media. This experiment was divided into six groups, including CMCM (group A), CMCM + 0.1 μM S1P (group B), CMCM + 0.5 μM S1P (group C), CMCM + 1.0 μM S1P (gorup D), DMEM + 5‐azacytidine (group E) and DMEM + 5‐azacytidine + 0.5 μM S1P (group F). The differentiations were examined by immunofluorescence staining of cardiac specific proteins, and the results were evaluated by confocal microscopy. All the differentiated cells in the six groups expressed three cardiac specific proteins (α‐actin, connexin‐43 and MYH‐6), but the fluorescence intensities from these expressions were different. The bars are 20 µm

**Figure 5:**
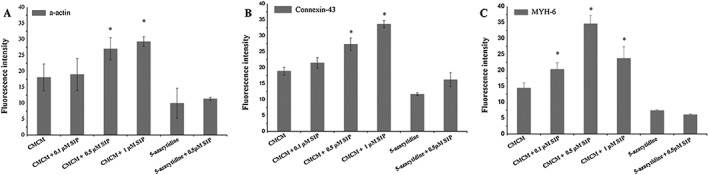
Fluorescence intensity analysis of the three cardiac specific proteins expressed in the six groups for hUCMSCs. Expressions of α‐actin and connexin‐43 were enhanced with increasing S1P concentration whilst that of MYH‐6 showed an initial increase with the concentration of S1P and then declined, reaching the peak value in CMCM + 0.5 μM S1P group. The fluorescence intensities were analysed using the software Image J. The data were expressed as mean ± SD (*n* = 3). Asterisk indicates statistically significant (*p* < 0.05) difference of fluorescence intensity compared with CMCM group or 5‐azacytidine group

The effects of S1P concentration on the cardiomyogenic differentiation of the two sources of MSCs are shown in Figures 3 and 4. For hUCMSCs, their α‐actin and connexin‐43 expressions increased with S1P concentration in CMCM groups (Figure 3), and there were significant increases compared with group A (CMCM) (Figure 5A, B; *p* < 0.05). In 5‐azacytidine groups, the expressions of α‐actin and connexin‐43 in the presence of S1P were stronger than those without S1P (Figure 3), but they were both lower than the culture conditions in CMCM without any 5‐azacytidine or S1P (Figure 5A, B), showing some subtle inhibitive influence from 5‐azacytidine to the function of S1P. Meanwhile, the expression of MYH‐6 increased with the concentration of S1P and reached the peak value in CMCM + 0.5 μM S1P group (Figure 3). Addition of S1P thus made significant differences compared with group A as in the case of the other two proteins (Figure 5C; *p* < 0.05). In contrast, addition of 5‐azacytidine with and without S1P in DMEM produced lower expressions of MYH‐6 than those in group A, a trend similar to those observed with the other two proteins, but there was no significant difference compared with group E (5‐azacytidine) (Figure 5C; *p* < 0.05).

**Figure 6:**
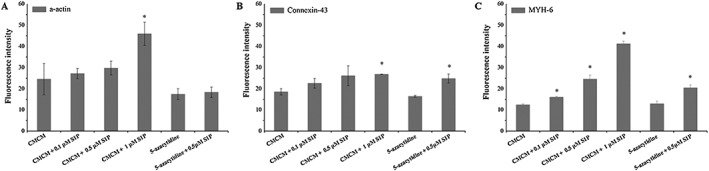
Fluorescence intensity analysis of the three cardiac specific proteins expressed in the six groups for hATMSCs. The expressions of the three cardiac specific proteins were all enhanced with increasing S1P concentration, whether in CMCM groups or in 5‐azacytidine groups. The fluorescence intensity was analysed using the software Image J. The data were expressed as mean ± SD (*n* = 3). Asterisk indicates statistically significant (*p* < 0.05) difference of fluorescence intensity compared with CMCM group or 5‐azacytidine group

For hATMSCs, whether in CMCM groups or 5‐azacytidine groups, the expressions of the three cardiac specific proteins were all enhanced by S1P and increased with its concentration (Figure 6), although the extent of increase varied and was different from the respective case as observed in hUCMSCs. In the CMCM groups, addition of 1 μM S1P led to substantial increases in α‐actin and MYH‐6. In the 5‐azacytidine groups, the levels of expressions of all three proteins were similar to their respective ones in group E (5‐azacytidine), but addition of 0.5 μM S1P led to increased expressions of conexion‐43 and MYH‐6 (Figure 6; *p* < 0.05).

### Influence of S1P concentration on cell activity

**Figure 7:**
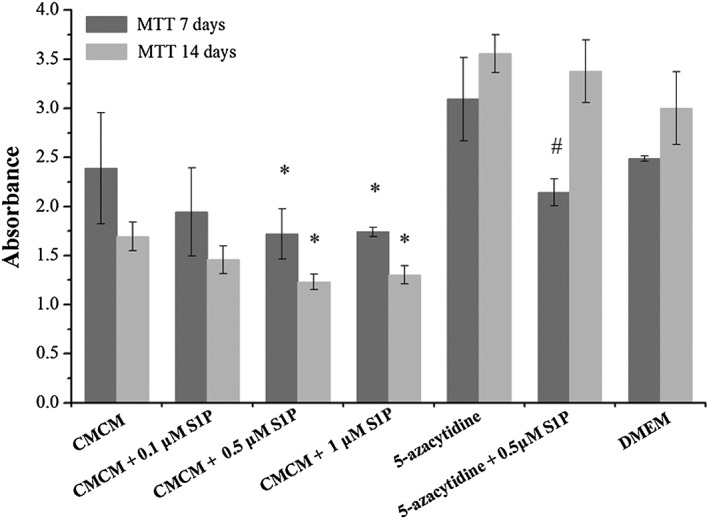
Cell activity assays in different groups of hUCMSCs by MTT. The experiment was divided into seven groups, including CMCM (group A), CMCM + 0.1 μM S1P (group B), CMCM + 0.5 μM S1P (group C), CMCM + 1.0 μM S1P (group D), DMEM + 5‐azacytidine (group E), DMEM + 5‐azacytidine + 0.5 μM S1P (group F) and DMEM (control group). Cell activities of hUCMSCs decreased with increasing S1P concentration according to the results of groups A–D on both 7th and 14th days. In groups A–D, the cell viability on the 14th day was lower than that on the 7th day. In group E and group F, S1P significantly reduced the cell viability. In group A and control group, cell activity cultured in DMEM was higher than that cultured in CMCM. The data were expressed as mean ± SD (*n* = 3). Aterisk indicates statistically significant (*p* < 0.05) difference of cell activity compared with CMCM group. Number sign indicates statistically significant (*p* < 0.05) difference of cell activity compared with 5‐azacytidine group

**Figure 8:**
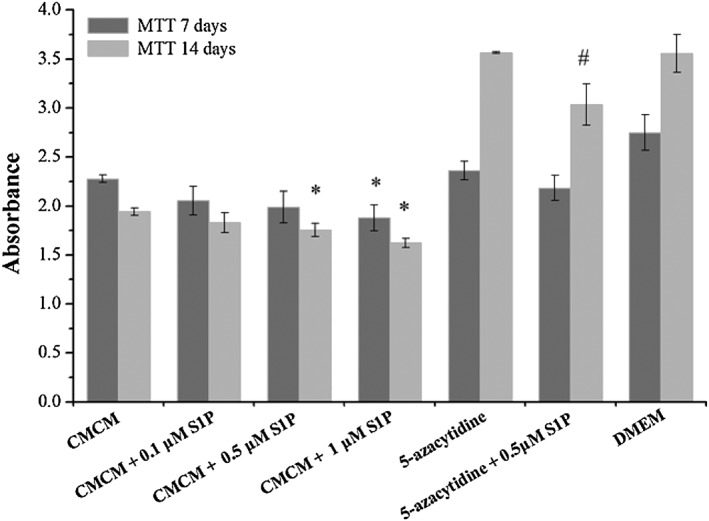
Cell activity assays in different groups of hATMSCs by MTT. The experiment was divided into seven groups, including CMCM (group A), CMCM + 0.1 μM S1P (group B), CMCM + 0.5 μM S1P (group C), CMCM + 1.0 μM S1P (group D), DMEM + 5‐azacytidine (group E), DMEM + 5‐azacytidine + 0.5 μM S1P (group F) and DMEM (control group). Cell activity of hATMSCs decreased with increasing S1P concentration according to the results of groups A–D on both 7th and 14th days. In groups A–D, the cell viability on the 14th day was lower than that on the 7th day. In groups E and F, S1P significantly reduced cell viability. In group A and control group, cell activity cultured in DMEM was higher than that cultured in CMCM. The data were expressed as mean ± SD (*n* = 3). Asterisk indicates statistically significant (*p* < 0.05) difference of cell activity compared with CMCM group. Number sign indicates statistically significant (*p* < 0.05) difference of cell activity compared with 5‐azacytidine group

The influences of S1P concentrations on cell activities of hUCMSCs and hATMSCs were assessed by MTT assays and are shown in Figures 7 and 8. For the CMCM series in groups A–D, cell activities decreased with time (as assayed on the 7th and 14th days) and decreasing S1P concentration. There were significant differences compared with the control (group A) (*p* < 0.05). In 5‐azacytidine groups (groups E and F), however, the trends are opposite as far as time and 5‐azacytidine are concerned, i.e. cell activities increased with time and addition of 5‐azacytidine, although the results did show the same trend of decrease of cell activity with addition of S1P (*p* < 0.05). In the DMEM systems (groups E and F) increase in cell viability with time because of DMEM acting as a popular cell growth medium. It was observed that cell activities cultured in DMEM were higher than those cultured in CMCM. CMCM is not a proliferation medium but a differentiation medium, hence explaining why the viabilities of the cells cultured in CMCM with or without S1P on the 14th day were lower than those on the 7th day.

### Effect of S1P on calcium transient

**Figure 9:**
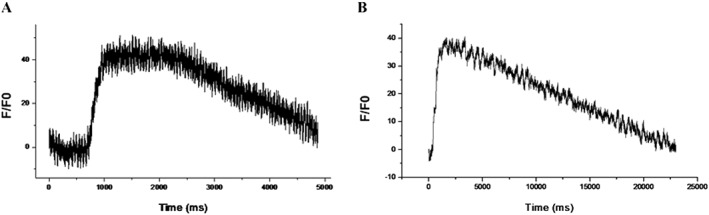
Calcium transient detections of differentiated cells in different groups. The electrophysiological features of the cells were assayed including group A (CMCM), group C (CMCM + 0.5 μM S1P), group E (5‐azacytidine) and group F (5‐azacytidine + 0.5 μM S1P). The results showed that calcium transients were observed only in hUCMSCs (A) or hATMSCs (B) in group C, while other groups did not express calcium transient (the data were shown in the Supporting Information)

Figure 9 shows the electrophysiological features of the cells from the four groups including group A (CMCM), group C (CMCM + 0.5 μM S1P), group E (5‐azacytidine) and group F (5‐azacytidine + 0.5 μM S1P). The results showed that the calcium transient happened only in the cells from group C, while the phenomenon could not be observed in other groups (data were shown in Figures S2 and S3 of Supporting Information).

## Discussion

MSCs are considered as sources of seed cells in tissue engineering, as they can be obtained from a wide variety of tissues, including bone marrow, adipose tissue, cord blood, placenta and peripheral blood.^19^, ^20^, ^21^ Recent studies have shown that umbilical cord and adipose tissue are two most attractive sources of MSCs^22^, ^23^, ^24^ as they are easy to obtain. This would change the current situation of treating them as medical wastes. A further benefit is that their future applications are not subject to strict ethical complications. Similar to MSCs from other sources, hUCMSCs and hATMSCs have the potential to differentiate into adipocytes, osteocytes, hepatocytes, fibroblasts and endothelial cells.^23^, ^25^, ^26^, ^27^, ^28^ In addition, hUCMSCs and hATMSCs can also differentiate into cardiomyocytes by treating them with 5‐azacytidine or maintaining them in CMCM.^6^, ^7^, ^29^, ^30^ In these studies, MSCs from the two sources were induced to differentiate into cardiomyocytes and expressed cardiac specific markers after 2 to 5 weeks, including sarcomeric α‐actinin, cardiac troponin I, β‐myosin heavy chain and connexin‐43. These cardiac specific markers enabled biological immunostaining as a powerful tool to investigate the role of the differentiations of hUCMSCs and hATMSCs into cardiomyocytes. We observed that hUCMSCs and hATMSCs consistently began to differentiate into cardiomyocytes and express cardiac specific markers around the 7th day of culturing. Our data showed that hUCMSCs and hATMSCs, whether treated with 5‐azacytidine or cultured with CMCM, began to differentiate into cardiomyocytes around 7 days of induction rather than widely reported longer time induction (from 2 to 5 weeks), an observation broadly consistent with previous work as reported by Zhao *et al.* 17 Furthermore, our work reported here also showed that the expressions of the three cardiac specific proteins at the 7th day were much weaker than those on the 14th or 28th days. This observation alone suggests that expressions of these specific proteins marking the extents of differentiations of hUCMSCs and hATMSCs into cardiomyocytes would need more than 2 weeks.

S1P has been shown to play a critical role in the regulation of numerous cardiovascular processes including angiogenesis, cardiac function, vascular development and cardiac development.^31^, ^32^, ^33^ In addition, a large number of studies have shown that S1P plays a key role in a number of fundamental biological processes including cell growth and survival, regulation of cell migration and differentiation.^14^, ^15^, ^34^ It is also implicated in the regulation of myogenic differentiation.^35^, ^36^ A recent study has suggested that S1P is able to maintain the multipotency of human adult bone marrow and adipose tissue‐derived stem cells.^37^ On the basis of these findings, we hypothesized that S1P might play roles in the differentiation of MSCs towards caridiomyocytes. In this study, we have not only confirmed that S1P promoted the differentiations of hUCMSCs or hATMSCs into functional cardiomyocytes when cultured in CMCM but also found that S1P could enhance the differentiations of hUCMSCs or hATMSCs into cardiomyocytes when induced with 5‐azacytidine. In addition, we found that the influence of S1P was concentration dependent, which was broadly in line with the report that S1P dose‐dependently stimulated the differentiation of hATMSCs towards smooth muscle cells.^13^ More importantly, we found that the differentiations were enhanced while cell activities decreased with S1P concentration increasing. There should thus be an optimal S1P concentration. On the basis of our data, we consider that 0.5 μM of S1P is the suitable concentration for the induction of hUCMSCs or hATMSCs to differentiate into cardiomyocytes.

This study has further demonstrated that S1P (in CMCM groups) not only induced expressions of characteristic proteins of cardiomyocytes but also gave rise to the calcium transients, one of the most important specific electrophysiological properties that rendered these cells capable of functioning *in vivo* as cardiomyocytes. On the other hand, no specific electrophysiological properties were observed in the 5‐azacytidine groups. This difference could be because of the cellular stimuli (e.g. transforming growth factor‐beta, TGF‐β) that may be contained in CMCM but not in 5‐azacytidine induction medium. S1P may stimulate MSCs to differentiate into functional cardiomyocytes by working synergistically with cellular factors (TGF‐β).^38^, ^39^

Until now, the mechanism of S1P on the cardiomyogenic differentiation of hUCMSCs or hATMSCs remains unclear. Several researchers have suggested that S1P influenced numerous physiological processes by coupling with G protein receptor family in the membrane.^15^, ^40^, ^41^ Wamhoff *et al.* demonstrated that S1P affected cell proliferation by regulating S1P_1_ and S1P_3_ receptors on cell surface.^42^ Additionally, S1P was found to play an important role in the differentiation of smooth muscle by S1P_2_ because its action was required in the expression of α‐actin.^43^ In another study, S1P_2_ and S1P_3_ were found to be involved in the differentiating action of S1P towards smooth muscle of progenitor mesodermal cells.^44^ Nincheri *et al.* 13 showed that hATMSCs could bear five different S1P receptors. And in these receptors, S1P_2_ was demonstrated to be, by pharmacological inhibition, the most important for transmitting the myogenic signal brought about by S1P, with a secondary role played by S1P_3_. Nevertheless, the exact mechanism of S1P working synergistically with cellular stimuli to affect the cardiomyogenic differentiation of hATMSCs or hUCMSCs deserves to be further investigated.

## Conclusions

From the consideration of expressions of the three cardiac specific proteins (α‐actin, connexin‐43 and MYH‐6) alone, culturing in both CMCM and 5‐azacytidine media would suffice in spite of variations in protein amount. S1P cannot only promote differentiations of hUCMSCs or hATMSCs into functional cardiaomyocytes when cultured in CMCM but also enhance their differentiations towards cardiomyocytes when working together with 5‐azacytidine. However, the benefit of addition of S1P with 5‐azacytidine varied. In CMCM, differentiations were enhanced with S1P concentration increasing, but cell activities declined. The suitable differentiation time was found to be around 14 days, and the optimal concentration of S1P was 0.5 μM in media. S1P in CMCM can also generate the calcium transients from the induced cardiomyocytes. Calcium transient is one of the most important specific electrophysiological properties for these cells to function *in vivo*, and the combinations of culturing conditions seemed to suggest that some optimal conditions might exist for promoting them to occur. Further work will be needed to establish these conditions.

## Conflict of Interest

The authors have declared that there is no conflict of interest.

## Supporting information

Supporting info item
