# Supplementary material for: Synergistic effect of bioactive lipid and condition medium on cardiac differentiation of human mesenchymal stem cells from different tissues
Source: Cell Biochem Funct. 2016 Mar 16;34(3):163–72. doi: 10.1002/cbf.3175 (PMC5031220; doi:10.1002/cbf.3175)

**Figure Legends**

Figure S1. The expressions of three cardiac specific proteins in control groups. hUCMSCs or hATMSCs were cultured in normal medium (DMEM) as negative control, HCM was cultured in the normal medium (DMEM) as positive control. Immunofluorescence staining showed that hUCMSCs (B) or hATMSCs (C) had little or no expression of the cardiac specific proteins (a-actin, connexin-43, and MYH-6), while HCM (A) clearly expressed the three cardiac specific proteins. The bars are 100 μm.

Figure S2. Calcium transient detections of differentiated cells for hUCMSCs in other groups. The calcium transients were not observed in group A (CMCM), group E (5-azacytidine), and group F (5-azacytidine + 0.5 µM S1P).

Figure S3. Calcium transient detections of differentiated cells for hATMSCs in other groups. The calcium transients were not observed in group A (CMCM), group E (5-azacytidine), and group F (5-azacytidine + 0.5 µM S1P).

Figure S1


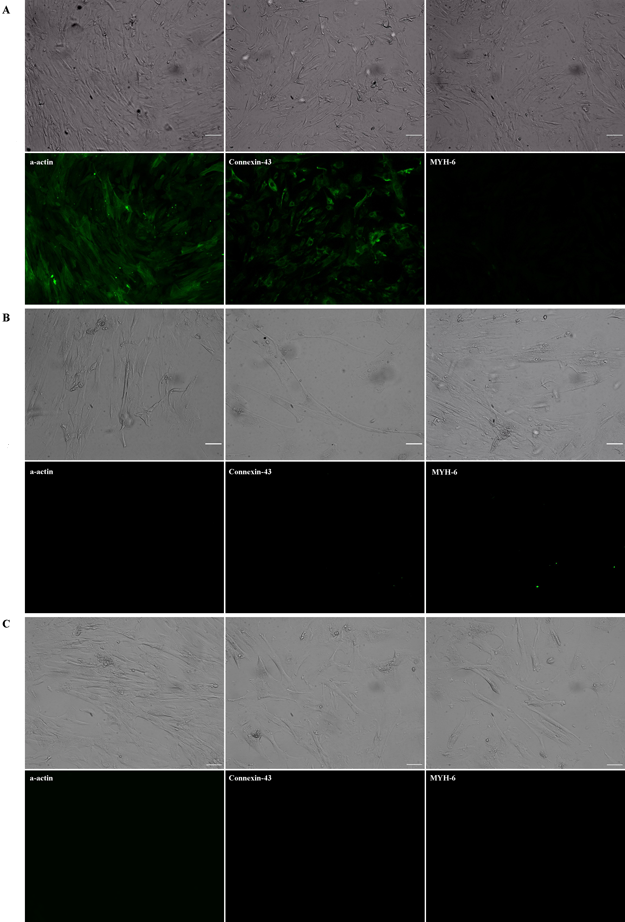


Figure S2


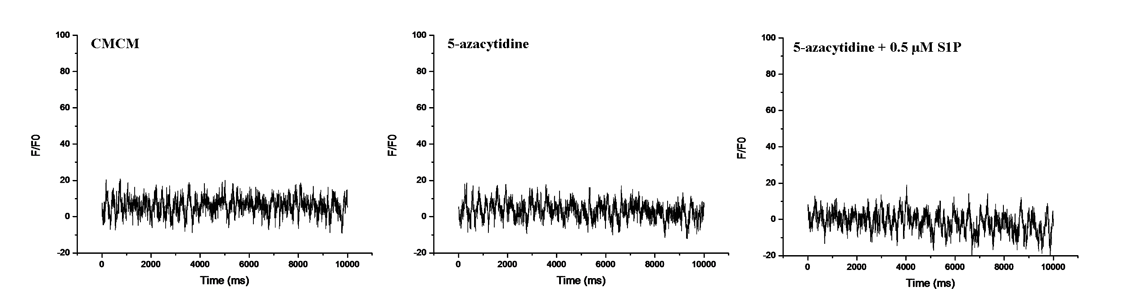


Figure S3


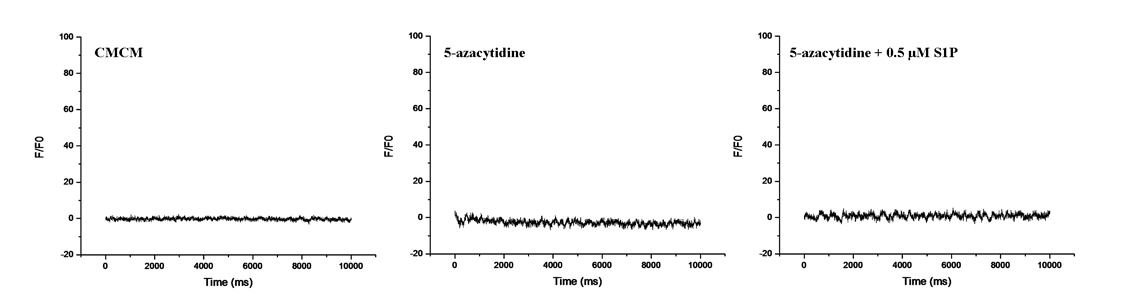

Supplement: Supplementary file 1 — Supporting info item [file CBF-34-163-s001.doc]
